# Phytochemicals of *Moringa oleifera*: a review of their nutritional, therapeutic and industrial significance

**DOI:** 10.1007/s13205-016-0526-3

**Published:** 2016-09-22

**Authors:** Ramesh Kumar Saini, Iyyakkannu Sivanesan, Young-Soo Keum

**Affiliations:** 1 Department of Bioresources and Food Science, College of Life and Environmental Sciences, Konkuk University, Seoul, 143-701 Korea; 2 Department of Molecular Biotechnology, College of Life and Environmental Sciences, Konkuk University, Seoul, 143-701 Korea

**Keywords:** Drumstick tree, Bioactives, Biodiesel, Water purification, *M. oleifera* cationic proteins (MOCP)

## Abstract

*Moringa oleifera* Lam., also known as the ‘drumstick tree,’ is recognized as a vibrant and affordable source of phytochemicals, having potential applications in medicines, functional food preparations, water purification, and biodiesel production. The multiple biological activities including antiproliferation, hepatoprotective, anti-inflammatory, antinociceptive, antiatherosclerotic, oxidative DNA damage protective, antiperoxidative, cardioprotective, as well as folk medicinal uses of *M. oleifera* (MO) are attributed to the presence of functional bioactive compounds, such as phenolic acids, flavonoids, alkaloids, phytosterols, natural sugars, vitamins, minerals, and organic acids. The low molecular weight of *M. oleifera* cationic proteins (MOCP) extracted from the seeds is very useful and is used in water purification, because of its potent antimicrobial and coagulant properties. Also, the *M. oleifera* methyl esters (MOME) produced from the oil of the seeds meet the major specifications of the biodiesel standard of Germany, Europe, and United States (US). Thus, MO is emerging as one of the prominent industrial crops for sustainable biodiesel production in tropical and subtropical countries. In view of the high nutritional, nutraceutical, and industrial values, it is important to compile an updated comprehensive review on the related aspects of this multipurpose and miracle tree. Hence, the present study is focused on the nutritionally significant bioactives and medicinal and biological properties, to explore the potential applications of MO in nutritionally rich food preparations. Furthermore, water coagulation, proteins, and fatty acid methyl esters from the MO seeds are reviewed, to explore their possible industrial applications in biodiesel production and water purification. In addition, the future perspectives in these areas are suggested.

## Introduction

The products derived from several herbs and plants, being a source of multifunctional curing agents and bioactive compounds, are relatively considered safe for consumption. According to the Food and Agriculture Organization’s (FAO) report, about 70–80 % of the world’s population, especially in developing countries, relies on herbal medicine to prevent and cure diseases (Ekor 2014), and about 25 % of the synthesized drugs are manufactured from medicinal plants (Pan et al. 2013). Increased demand for food to tackle hunger and malnutrition problems has been pertinent in developing countries over the last few decades. In Asian and African countries, the vast majority of the population suffers from malnutrition because of the deficiency of essential nutrients in foods. *Moringa oleifera* Lam. (syn. *M. pterygosperma* Gaertn., 2*n* = 28) belongs to the family Moringaceae, commonly known as the ‘drumstick’ or ‘horseradish’ tree. It is an affordable and readily available source of major essential nutrients and nutraceuticals, and it has the potential to eradicate malnutrition (Kunyanga et al. 2013). The *Moringa* is often considered as important famine food because of its high resistance to drought and arid conditions owing to their tuberous roots (Padayachee and Baijnath 2012). Almost each and every part of *Moringa* tree is useful for medicinal, functional food preparations, nutraceuticals, water purification, and biodiesel production; including roots, leaves, flowers, green pods, and seeds (Saini 2015). The immature pods, flowers, and foliage of this tree are used for culinary purposes in different parts of the world (Stevens et al. 2013). The foliage of *M. oleifera* (MO) has been established as a rich source of phenolics and glucosinolates (Amaglo et al. 2010), minerals (Saini et al. 2014a), tocopherols (Saini et al. 2014b), carotenoids (Saini et al. 2014c), polyunsaturated fatty acids (Saini et al. 2014d), ascorbic acid (Saini 2015), and folate (Saini et al. 2016). *Moringa* seed oil (yield 30–40 % w/w), also known as “Ben oil” is used for the production of biodiesel, because of the high content of monounsaturated fatty acids in the form of oleic acid (C18:1) (Azam et al. 2005; Rashid et al. 2008). *Moringa* seed oil is a potential candidate for biodiesel production, as it meets all the main specifications of the biodiesel standards of US, Germany, and Europe (Mofijur et al. 2014). Thus, it has great commercial and industrial importance. The low molecular weight cationic proteins (MOCP), chitin binding protein isoforms (Mo-CBP3), lectins, napins, mabinlins, and other seed proteins extracted from MO seeds are successfully characterized and used in domestic and industrial water purification, and hardness removal because of the potent antimicrobial and coagulant properties (Kansal and Kumari 2014).

To our knowledge, no recent comprehensive reviews exist on water coagulation proteins and its coagulation and flocculation mechanism. Also, a significant amount of work has been conducted to explore the potential of MOME in biodiesel production. Thus, after reviewing the literature on nutritional, nutraceuticals, water purification and biodiesel potential uses of MO, it is worthwhile to compile an updated and comprehensive review. Thus, the present study is focused on the bioactive composition, medicinal and biological properties, water coagulation proteins, and fatty acid methyl esters from different parts of MO, to explore its sustainable uses in the domestic and industrial sectors. Additionally, future perspectives in these contexts are identified.

### Botanical description, distribution, and production

**Fig. 1:**
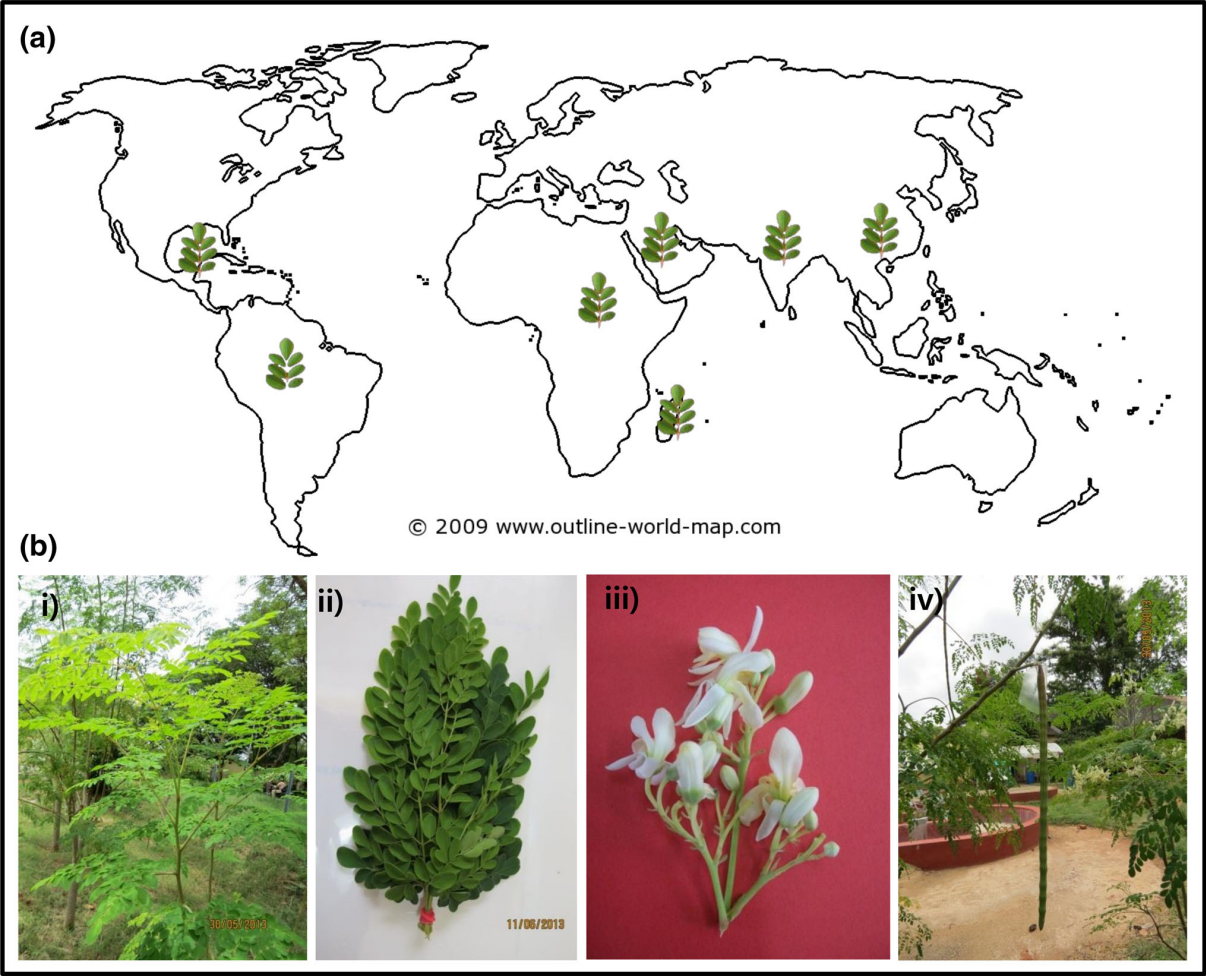
**a** The distribution of *Moringa oleifera* in the World. The image of world map was obtained from www.outline-world-map.com (royalty free). **b** Different vegetative and reproductive parts of *M. oleifera* tree; *i* field grown tree, *ii* bundle of foliage, *iii* flowers, and *iv* fruit (pod)

Moringaceae is a single genus family of shrubs and trees, which comprise of 13 species, distributed in the Indian subcontinent (*M. oleifera* and *M. concanensis*), Kenya (*M. longituba* and *M. rivae*), northeastern and southwestern Africa (*M. stenopetala*), Arabia, and Madagascar (*M. drouhardii* and *M. hildebrandtii*) (Padayachee and Baijnath 2012; Saini 2015). *Moringa oleifera* Lam. is a tropical deciduous perennial dicotyledonous tree. The stem is brittle with a corky, whitish-gray bark, with drooping branches, pale green and bipinnate or more commonly tripinnate leaves (30–60 cm long) with opposite, ovate leaflets (Pandey et al. 2011). *M. Oleifera,* the native of the sub-Himalayan mountains of northern India; is now cultivated for a variety of purposes in the whole tropical and sub-tropical regions of the world (Leone et al. 2015). The distribution of *M. oleifera* in the world is outlined in Fig. 1a. Similarly, different vegetative and reproductive parts of *M. oleifera* tree is shown in Fig. 1b. Zaku et al. (2015) recently reviewed the potential of the MO tree, emphasizing its nutritional applications for humans and industrial uses, and also described its propagation methods. It is propagated through cuttings (0.2–1.0 m long), with recommended tree to tree spacing of 1.2 and 5 m between rows (for pod yield), to obtain the desirable population of 1666 trees/ha. For foliage production, cuttings are planted with a close spacing to obtain≈1 million trees/ha. Propagation through seeds is not recommended because of substantial genetic variation through cross-pollination (Saini et al. 2013). The *Moringa* tree grows best in the temperature range of 25–35 °C, under direct sunlight, at an altitude of 500 m, and in slightly acidic to alkaline soil (pH 5.0–9.0); although it can tolerate excess temperature, up to 48 °C, frost in winter, altitude, and a wide variety of soil conditions. MO seeds can be planted just after maturity, as the seeds do not undergo dormancy while retaining viability up to 1 year. The tree starts bearing fruits at an age between six and 8 months, with a low fruit set in the initial one to 2 years, however, the yield increases in the subsequent years. The productivity of the Brazilian genotype was estimated as 45 tons of pods per hectare (da Silva et al. 2010). The oil yield of 258 kg/ha was recorded from the Indian cultivar (PKM-1), grown in the subtropical north-western region of Argentina, after 3 years of the plantation (Ayerza 2011). India is the largest producer of MO fruits (pods) with an annual production of 1.1–1.3 million tons from an area of 38,000 ha. In India, the state of Andhra Pradesh is the major producer both area-wise (15665 ha) and in production, followed by Karnataka (10280 ha) and Tamil Nadu (7408 ha) (Patel et al. 2010).

### Phytochemical composition

Different parts of the MO tree have been established as being good sources of unique glucosinolates, flavonoids and phenolic acids (Amaglo et al. 2010; Coppin et al. 2013), carotenoids (Saini et al. 2014c), tocopherols (Saini et al. 2014e), polyunsaturated fatty acids (PUFAs) (Saini et al. 2014d), highly bioavailable minerals (Saini et al. 2014a), and folate (Saini et al. 2016). Among glucosinolates, 4-O-(a-L-rhamnopyranosyloxy)-benzylglucosinolate (glucomoringin) is the most predominant in the stem, leaves, flowers, pods and seeds of *M. oleifera* (Amaglo et al. 2010). Although in the roots, benzyl glucosinolate (glucotropaeolin) is the most prominent. The highest content of glucosinolate is found in the leaves and seeds. The enzymatic catabolism of glucosinolates by the endogenous plant enzyme myrosinase produces isothiocyanates, nitriles, and thiocarbamates that are known for strong hypotensive (blood pressure lowering) and spasmolytic (muscle relaxant) effects (Anwar et al. 2007). Among flavonoids, flavonol glycosides (glucosides, rutinosides, and malonyl glucosides) of quercetin > kaempferol > isorhamnetin are predominantly found in various parts of the tree, except in the roots and seeds. In the leaves, the amount of quercetin and kaempferol was found to be in the range of 0.07–1.26 and 0.05–0.67 %, respectively. Also, among different varieties, the Indian varieties (PKM-1 and PKM-2) have shown a higher total content of quercetin and kaempferol, compared to the African indigenous samples (Coppin et al. 2013). The potent antioxidant activity MO is attributed to the high concentration of these polyphenols. Of late, seven major cultivars of MO from Pakistan have been characterized for their polyphenolic, nutrient, and antioxidant potential. The quercetin, apigenin, and kaempferol derivatives were recorded; the major flavonoids in the hydromethanolic extracts of the *Moringa* foliage, corresponded to 47.0, 20.9, and 30.0 % of the total flavonoids (on an average), respectively. The varying concentrations of phenolics with the antioxidant capacity of the tested foliage established ‘Pakistan Black’ and ‘Techiman’ as the most nutritive cultivars, compared to the other major cultivars of MO from Pakistan (Nouman et al. 2016).

5-Formyl-5,6,7,8-tetrahydrofolic acid (5-HCO-H_4_folate; 502.1 μg/100 g DW), 5,6,7,8-tetrahydrofolic acid (H_4_folate; 223.9 μg/100 g DW), 5-Methyl-5,6,7,8-tetrahydrofolic acid (5-CH3-H_4_folate; 144.9 μg/100 g DW), and 10-Formylfolic acid (10-HCO-folic acid; 29.0 μg/100 g DW) are the major forms of folates found in the foliage of MO (Saini et al. 2016). Additionally, these forms are highly bioavailable in animals, compared to other folate-rich foods, such as green leafy vegetables. Relative bioavailability, calculated as the response of *Moringa* folates compared to the response of synthetic folic acid in a rat model, was recorded as 81.9 %. In the calculations of the recommended dietary allowances (RDA), only 50 % of natural folate is assumed to be bioavailable. Thus, it is suggested that MO-based food can be used as a significant source of folate, because of significantly higher bioavailability in animals. Folate is the one of the most important water-soluble vitamins, plays an essential role in various cellular metabolisms, including oxidation and reduction of one-carbon units (Scotti et al. 2013). Folate deficiency causes severe chronic diseases and developmental disorders, including neural tube defects (NTDs) during pregnancy (Williams et al. 2015). Thus, a folate-sufficient diet is strongly recommended during pregnancy to prevent the NTDs and other chronic dysfunctions.

The foliage, flowers, and immature pods (fruits) of various commercially grown Indian cultivars of MO have been characterized by the content of carotenoids (Saini et al. 2014c). All-*E*-lutein is the major carotenoid in foliage and immature pods (fruits), accounting for 53.6 and 52.0 % of the total carotenoids, respectively. Other carotenoids, such as, all-*E*-luteoxanthin, 13-*Z*-lutein, all-*E*-zeaxanthin, and 15-*Z*-β-carotene have also been found in minor quantities. Among various tissues, the highest content of total carotenoids is recorded in leaves (44.30–80.48 mg/100 g FW), followed by immature pods (29.66 mg/100 g FW), and flowers (5.44 mg/100 g FW). Among the various Indian cultivars, the highest content of all-*E*-zeaxanthin, all-*E*-β-carotene, and total carotenoids was recorded in the Bhagya (KDM-1) cultivar (Saini et al. 2012, 2014c). The MO leaves are a rich source of α-tocopherol (vitamin E), accounting for 17.3 mg/100 g FW in the PKM-1 cultivar. With evidence from various studies, the foliage of MO is established as a rich source of carotenoids and tocopherols. However, these vitamins are significantly degraded during dehydration and the other processes that occur in the *Moringa* foliage (Saini et al. 2014e). Thus, experiments have also been conducted to further enhance the content of these vitamins in the foliage of MO (Saini et al. 2014b), and interestingly, foliar administration of biotic elicitors (carboxy-methyl chitosan and chitosan) and signaling molecules (methyl jasmonate and salicylic acid) has been found to be potentially beneficial for the enhancement of major carotenoids and α-tocopherol in the foliage of field-grown MO trees. Elicitation with 0.1 mM salicylic acid (SA) has been found to accumulate 49.7 mg/100 g FW of α-tocopherol, which represents a 187.5 % increase, compared to the untreated control. Thus, there is an excellent perspective for enhancement of these vitamins in the foliage that can be useful for improving the nutraceutical benefits of this tree.

The MO leaves are also established as a rich source of omega-3 (ω-3) and omega-6 (ω-6) polyunsaturated fatty acids (PUFAs), in the form of α-linolenic acid (C18:3, ω-3, 49–59 %), and linoleic acid (C18:2, ω-6, 6–13 %). Palmitic acid (C16:0) is recorded in the major saturated fatty acid, accounting for 16–18 % of the total fatty acids in the *Moring*a leaves. Immature pods and flowers are characterized by a higher content of total monounsaturated fatty acids (MUFAs, 16–30 %) and are low in PUFAs (34–47 %), compared to the leaves (Saini et al. 2014d). In contrast, the seeds and seed oil have a high content of oleic (18:1, 70–80 %), palmitoleic (16:1, 6–10 %), stearic (18:0, 4–10 %), and arachidic acid (20:0, 2–4 %), and a lower content of oleic, linoleic, and linolenic acid (Amaglo et al. 2010). This seed oil contains an identical fatty acid profile such as olive oil except for linoleic acid (Sánchez-Machado et al. 2015). To obtain the highest yield of oil from seeds, the solvent-assisted extraction using chloroform and methanol in the ratio of 3:1 at 100 °C is seen to be most favorable. However, oil extracted with these solvents is not recommended for human consumption because of the residual amount of these toxic substances. Thus, hexane is routinely used in the extraction of oil from *Moringa* seeds, because of its efficiency and ease of recovery. The thermogravimetric analysis (TGA) analysis revealed that the oil degrades at a temperature of about 425–450 °C (Bhutada et al. 2016). In terms of health effects, the *M. oleifera* leaves, immature pods, flowers, seeds, and seed oil have a low saturated fatty acid (SFAs) content and high MUFA and PUFA content that can enhance the health benefits of *Moringa*-based foods. The details of fatty acids from seeds are given in the methyl esters (Biodiesel) section.

Potassium (K), calcium (Ca), and magnesium (Mg) are the predominant minerals in the MO tissues. The highest content of K is found in the vegetative parts and immature pods, whereas, leaves and seeds are a rich source of Ca and Mg, respectively (Amaglo et al. 2010). MO is also recorded as having a rich source of iron (Fe) (17.5 mg/100 g DW). In a bioavailability study conducted on a rat model, Fe from the *Moringa* leaf was found to be superior compared to ferric citrate, in overcoming iron deficiency (Saini et al. 2014a). Significant changes in the expression (up to 100-fold) of liver hepcidin (HAMP) and other liver iron-responsive genes are also recorded in response to the Fe deficiency, suggesting that the relative expression of liver hepcidin (HAMP) mRNA can be used as the most sensitive molecular marker to detect iron-deficiency in animals. The results of animal bioavailability studies suggest that *Moringa* foliage can be used as a significant source of iron, because of its significantly higher bioavailability.

Full-fat and defatted MO kernels are recorded as being rich in protein content and account for 36.18 and 62.76 %, respectively. The concentrations of the other proximate constituents were found to be higher in defatted flour, compared to full-fat flour. Defatting also increased water absorption, fat absorption, foaming capacity, and foam stability of flour (Ogunsina et al. 2010). The author suggested that the MO kernel flour could be used as a valuable source of protein in food product formulation. In the proximate studies from Brazil, the dehydrated leaf powder was recorded to contain 44.4 % carbohydrate, 28.7 % crude protein, 10.9 % ash, 7.1 % fat, 103.1 mg/100 g iron, and 3.0 mg/100 g calcium. Similarly, the protein profile showed 70.1 % insoluble proteins, 3.5 % glutelin, 3.1 % albumin, 2.2 % prolamin, and 0.3 % globulins. Antinutritional compounds, such as, tannins (20.7 mg/g), trypsin inhibitor (1.45 TIU mg/g; Trypsin Inhibitor Units), nitrates (17 mg/g), and oxalic acids (10.5 mg/g) were also documented (Teixeira et al. 2014).

(Oyeyinka and Oyeyinka 2016), recently reviewed the possibilities of food fortification with MO leaf, seed, and flower powder to improve the nutritional value. Authors describe the fortification possibilities in various staple foods such as Amala (stiff dough), ogi (maize gruel), bread, biscuits, yogurt, and cheese for making soups. Authors described that although many of the reviewed studies reported improvement in the nutritional value of staple foods fortified with MO, none of the reports showed the in vivo or in vitro digestibility and availability of nutrients. Thus, the nutrient bioavailability and phytochemical contents of MO-fortified foods should be determined.

### Biological activity and therapeutic potential of *M. oleifera*

The different parts of the MO tree, including roots, bark, leaves, flowers, fruits, and seeds are traditionally used in various therapeutic applications, including, abdominal tumors, hysteria (a psychological disorder), scurvy, paralysis, helminthic bladder, prostate problems, sores and other skin infections. The therapeutic potential and medicinal properties of MO are extensively reviewed (Farooq et al. 2012; Mbikay 2012). Stohs and Hartman (2015) described the physiological and pharmacological activities of the leaves, seeds, bark, roots, sap, and flowers of *M. oleifera.* The various safety studies conducted on animals are also reviewed by the authors and they have concluded that the *Moringa* foliage, flower, and fruit extracts offer a high degree of safety without any adverse effects on humans. The phytochemicals of MO have shown antidyslipidemic, anthelmintic, antihyperglycemic, anti-inflammatory, antimicrobial, antioxidant, antiproliferative, anti-ulcer, antiurolithiatic, and hepatoprotective properties (Tables 1, 2). Potent antiproliferative and apoptotic properties of the MO leaf extract, rich in quercetin and kaempferol phenolics compounds, have been demonstrated using the human tumor (KB) cell line model. The MO leaf extract has shown significant morphological changes and decreased cell viability, with increased internucleosomal DNA fragmentation and ROS generation in the KB cells (Sreelatha et al. 2011).

**Table 1:** Biological activities of different extracts of *M. oleifera*

Extract	Activities	References
Water extract of foliage	Hypolipidemic and antiatherosclerotic	Chumark et al. (2008)
Aqueous extract of foliage, fruits, and seeds	Oxidative DNA damage protective	Singh et al. (2009)
Hydroalcoholic extract of foliage	Antiperoxidative and cardioprotective	Nandave et al. (2009)
Water extract of foliage	Antiproliferation and apoptosis cancer cells	Sreelatha et al. (2011)
Hydroethanolic extracts of foliage, fruits, and seeds	Hepatoprotective	Fakurazi et al. (2012), Ujah et al. (2013)
Methanol and ethanol extract of foliage	Inhibition of differentiation of colon cancer cells	Lea et al. (2012)
Aqueous extract of foliage	Down-regulation of nuclear factor-kappaB	Berkovich et al. (2013)
Ethanol extract of foliage	Upregulation of TNF-α	Akanni et al. (2014)
Ethanol extract of foliage	Hypolipidaemic in Hypercholesterolemic Rats	Atsukwei et al. (2014)
Methanol extract of foliage	Antioxidant, anti-inflammatory and antinociceptive	Adedapo et al. (2014)
Acetone extracts of foliage	Antimicrobial and antioxidant	Ratshilivha et al. (2014)
Methanol, ethyl acetate, and aqueous extracts of seeds	Antimicrobial	Emmanuel et al. (2014)
Isothiocyanate-rich extract of foliage	Weight gain, insulin resistance and hepatic gluconeogenesis	Waterman et al. (2015)
Ethanol extract of foliage	Antihyperglycemic and hypolipidemic	Irfan et al. (2016)
Aqueous extract of foliage	Immunity against Herpes Simplex Virus Type 1 (HSV-1)	Kurokawa et al. (2016)

**Table 2:** Antimicrobial activity of different extracts of *M. oleifera*

Extract type	Microorganisms	References
Ethanol extracts of seeds and foliage	*Trichophyton rubrum, Trichophyton mentagrophytes, Epidermophyton floccosum,* and *Microsporum canis*	Chuang et al. (2007)
Chloroform and ethanol extracts of seeds and foliage	*Escherichia coli, Pseudomonas aeruginosa, Staphylococcus aureus* and *Enterobacter aerogenes*	Bukar et al. (2010)
Water-soluble seed lectin	*Staphylococcus aureus* and *Escherichia coli*	Ferreira et al. (2011)
Phenolics rich extracts of seed flour	*Bacillus cereus, Staphylococcus aureus, Escherichia coli* and *Yersinia enterocolitica*	Govardhan Singh et al. (2013)
Hexane, petroleum ether, butanol, chloroform, acetone, ethyl acetate, methanol, and water extract of pod husks	*Staphylococcus aureus, Staphylococcus epidermidis, Salmonella typhimurium, Escherichia coli,* and *Klebsiella pneumoniae*	Arora and Onsare (2014)
Acetone extracts of foliage	*Candida albicans*, *Aspergillus fumigatus*, *Cryptococcus neoformans, Staphylococcus aureus*, *Enterococcus faecalis*, *Escherichia coli*, and *Pseudomonas aeruginosa*	Ratshilivha et al. (2014)
Methanol, ethyl acetate, and aqueous extracts of seeds	*Escherichia coli*, *Klebsiella pneumonia*, *Proteus mirabilis*, *Pseudomonas aeruginosa* and *Staphylococcus aureus*	Emmanuel et al. (2014)
Flavonoids extracts of seeds	*Staphylococcus aureus, Pseudomonas aeruginosa* and *Candida albicans*	Onsare and Arora (2015)

Chronic hyperglycemia is an indicator of diabetes mellitus (Type-2 DM), similarly, chronic dyslipidemia is a potential risk factor for cardiovascular disease (CVD). In animal studies, *Moringa* leaf water extract is found to control the fasting plasma glucose levels (FPG), postprandial blood glucose (PPPG), Blood glycated hemoglobin (HbA_1c_), and increased glucose tolerance, studied by the oral glucose tolerance test (OGTT), in streptozotocin (STZ)-induced diabetic rats (Jaiswal et al. 2009), and untreated T2DM patients (Arun Giridhari et al. 2011). The *Moringa* leaf water and methanol extract are also reported to have antidyslipidemic effects to lower the serum total cholesterol (TC), triacylglyceride (TG), very low-density lipoprotein (VLDL), low-density lipoprotein (LDL), and the atherogenic index, with increased high-density lipoprotein (HDL) in rats fed on a high-fat diet (HFD). A significant rise in the fecal excretion of cholesterol is observed in treated animals compared to the HFD control group (Jain et al. 2010). Similar antidyslipidemic effects are also documented in hyperlipidemic patients (TC > 180 mg/dL and TG > 140 mg/dL), fed with leaf tablets (4.6 g/day) for 40–50 days (Nambiar et al. 2010).

The MO aqueous leaf extract downregulates a pro-inflammatory transcription factor (nuclear factor-kappa B; NF-kB) and increases the cytotoxic effect in apoptosis-based chemotherapy, investigated in cultured human pancreatic cancer cells (Panc-1, p34, and COLO 357). The treatment of the extract (≥0.75 mg/ml) induces a rise in the sub-G1 cell populations of the cell-cycle and reduces the expression of different subunits of NF-kB, namely, p65, p-IkBα, and IkBα. Also, the leaf extract synergistically enhances the cytotoxic effect of cisplatin on Panc-1 cells (Berkovich et al. 2013). MO aqueous leaf extract containing 1.66 % isothiocyanates [4-(α-L-rhamnosyloxy)- and 4-(4′-O-acetyl-α-L-rhamnosyloxy)-benzyl isothiocyanate], and 3.82 % total polyphenols, significantly decreases gene expression and production of inflammatory markers (*iNOS* and *IL*-*1β*) in RAW macrophages. These results suggest that concentrated MO isothiocyanates can be used to alleviate low-grade inflammation associated with chronic diseases (Waterman et al. 2014). An alkaloid compound, N, α-L-rhamnopyranosyl vincos amide (VR) extracted from leaves of MO has shown the protection against isoproterenol (ISO)-induced cardiac toxicity in rats. The oral administration of VR at 40 mg/kg of body weight for 1 week significantly reduces an ISO-induced upsurge in the levels of the serum cardiac markers, such as, troponin-T (cTnT), creatine phosphokinase-MB (CK-MB), glutamate pyruvate transaminase (SGPT), lactate dehydrogenase (LDH), as well as, cardiac lipid peroxidation, with an increase in cellular antioxidants (Panda et al. 2013). The MO leaves are also a rich source of phenolics and flavonoids and exhibit potent antioxidant activity both in in vitro and in vivo systems. The highest extractability of total phenolics (13.23 g chlorogenic acid equivalents/100 g extract) and total flavonoids (6.20 g isoquercitrin equivalents/100 g extract) is obtained with 70 % ethanol. This extract has exhibited a high DPPH-scavenging activity (EC_50_ of 62.94 μg/mL) and the highest ferric reducing power (FRP) (51.50 mmol FeSO4 equivalents/100 g extract). Also, at the concentration of 100 μg/mL, the extract is seen to reduce a relative amount of intracellular ROS significantly (Vongsak et al. 2013).

### Water coagulation proteins from *M. oleifera* seeds

Turbidity is caused by suspended negatively charged particles and natural organic matter present in the water. On account of the surface electrical charge, these particles mutually repel each other, making it difficult for them to aggregate and settle. Thus, to overcome the repulsive charge and “destabilize” the suspension, a coagulant with the opposite charge is added to the water. Aluminium, Fe^+3^ salts and synthetic polymers are the most widely used coagulants in turbid and wastewater treatment (Kansal and Kumari 2014). However, there are several serious drawbacks of using synthetic chemicals and polymers because of the harmful effects on human health and the environment. Thus, a revival of interest in natural coagulants has emerged because of the high-cost factor, health-related issues, and the environmental impacts associated with synthetic coagulants (Choy et al. 2014). Seeds of *M. oleifera*, *Vigna unguiculata*, *Parkinsonia aculeata*, and more recently, an endosperm of *Cocos nucifera* are used as prospective natural coagulants (Choy et al. 2014). Among all these natural coagulants, proteins extracted from *M. oleifera* seeds have received significant attention in water purification, because of potent antimicrobial and flocculant properties. A large number of active proteins with flocculating properties have been isolated and extracted and characterized from *Moringa* seeds. In the first report, a 6.5 kDa cationic protein (MO_2.1_ and MO_2.2_; Swissprot, P24303), with isoelectric the isoelectric points above pH 10, has been characterized from *Moringa* seeds. The amino acid sequencing showed high contents of glutamine, arginine, and proline, with a total of 60 residues (Gassenschmidt et al. 1995). This cationic protein is commonly known as *Moringa oleifera* cationic protein (MOCP, also referred to as Flo), which actively inhibits bacterial cell growth and settles negatively charged particles in a solution. Analysis of an MOCP amino acid sequence has indicated a significant similarity with the 2S albumin seed storage protein family (napins and mabinlins), which are the most abundant storage proteins found in plant seeds (Suarez et al. 2003). Plant 2S albumin proteins are primary sources of carbon and nitrogen and are involved in plant defense. A 30 kDa thermal resistant coagulant lectin (cMoL), which is active at a pH range of 4.0–9.0, and stable at 100 °C is also identified from *Moringa* seeds; and possesses potent water coagulant properties (Santos et al. 2009). Following this, primary sequence studies have revealed a molecular mass of 11.928 kDa of this protein. These results suggest that cMoL is a trimer consisting of three subunits of 11.928 kDa (Luz et al. 2013). The cationic nature of cMoL is because of high contents of glutamine > alanine > proline, and positively charged amino acids (arginines and histidines). The secondary structure of cMoL at 7.0 pH has been estimated as 46 % α-helix, 12 % β-sheets, 17 % β-turns, and 25 % unordered structures belonging to the α/β tertiary structure class. Of late, the chitin-binding protein isoform (Mo-CBP_3_), a 2S albumin protein, is being extracted and characterized from *Moringa* seeds and it possesses potent antifungal, antibacterial, and flocculating activities (Freire et al. 2015; Ullah et al. 2015).

The coagulation efficiency of MOCP is stable during storage temperature; however, the coagulation efficiency decreases as the storage duration increases. Interestingly, the coagulation efficiency of MOCP is found to be increased with an increase in the initial turbidity of water (Katayon et al. 2006). The key structural features of MOCP attribute its functionality as an antimicrobial agent. MOCP is a cationic protein and contains a net positive charge that facilitates the interaction with microbial anionic lipid membranes. MOCP also possesses an amphiphilic helix-loop–helix motif that helps it to integrate into the bacterial membranes. Owing to these functional properties, MOCPs are selectively targeted and kill several microbes, including waterborne pathogens. It has been established that membrane fusion is the most dominant mechanism of MOCP antimicrobial activity (Shebek et al. 2015). Of late, solvent-defatted seed extracts have shown an improved water coagulant efficiency. Solvent-defatted seed extracts show a coagulant efficiency comparable to that observed for polyaluminium chloride (PAC) coagulant (≈88 %), while non-defatted seed extract efficiency was 30 % lower. Additionally, Soxhlet extraction with ethanol (for oil extraction) allows obtaining a defatted extract that requires doses of coagulant protein 5–33 times lower, compared to hexane or acetone defatted extract. Thus, the authors recommend defatting of the extract using ethanol before actual extract preparation to achieve the maximum coagulant efficiency, with lower doses of protein.

### The mechanism of water coagulation and flocculation

**Fig. 2:**
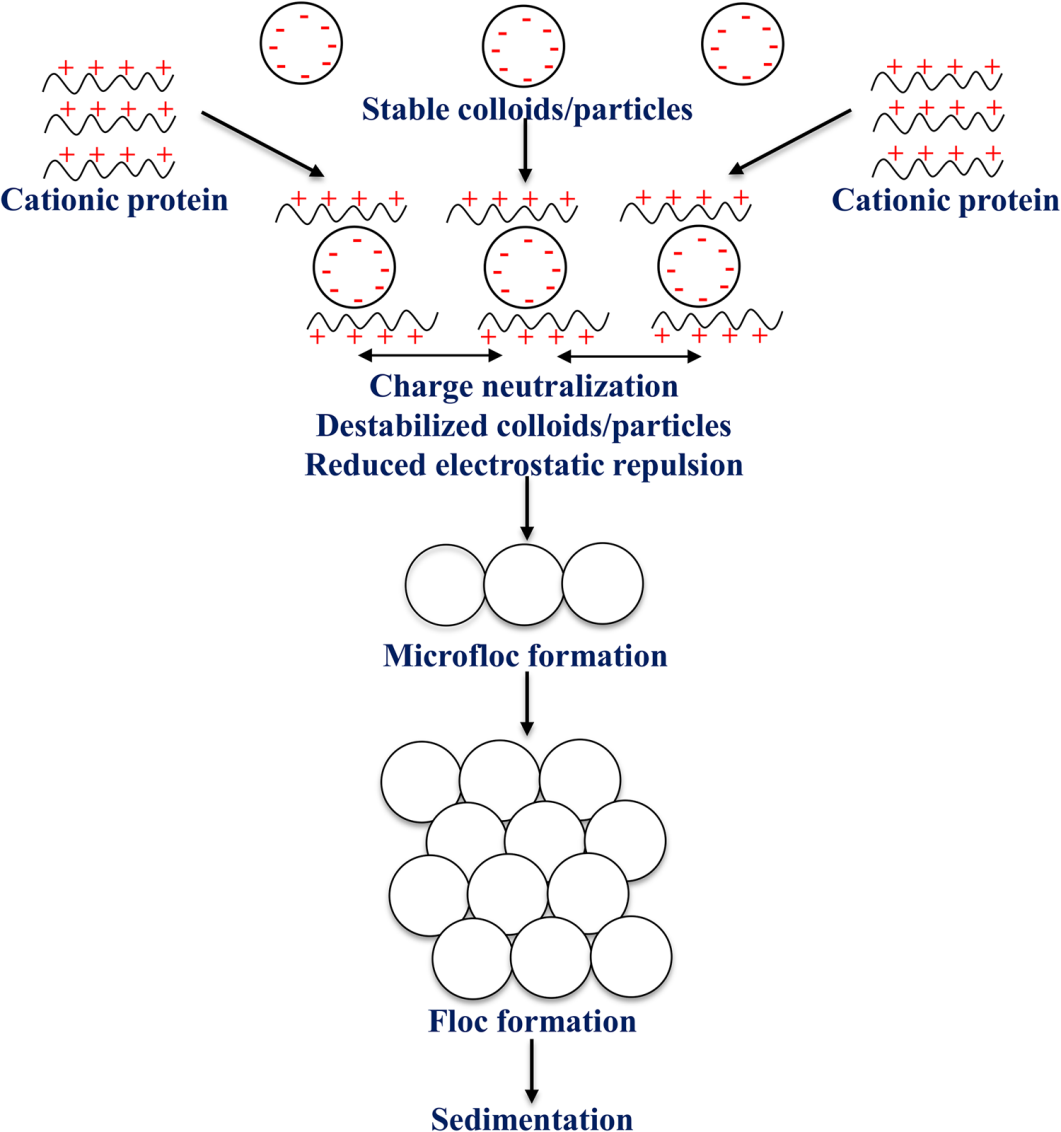
Mechanism of water coagulation and sedimentation using *Moringa oleifera* cationic proteins (MOCP)

The large numbers of the water coagulation mechanisms have proposed MOCP. Among them, adsorption and charge neutralization, and adsorption and bridging of destabilized particles are most accepted as the primary coagulation mechanisms (Kansal and Kumari 2014). These two mechanisms may be taking place simultaneously. In the studies of (Gassenschmidt et al. 1995) the coagulation efficiency of MOCP is comparable with a synthetic cationic polymer 554 K. The flocculent activity of a high molecular cationic polymer like 544 K was explained by the ‘bridge formation model’. The flocculation of the negatively charged particles (impurities) is a result of the binding of positively charged macromolecules (cationic polymer) to the surfaces of particles by Coulomb forces. The neutralization of part of the surface charge and reduction in the electrostatic repulsion leads to agglomeration of particles. Meanwhile, only a small part of the charged macromolecule binds to the surface of one particle, even as the major portion is free to bind to the surface of another particle. This leads to agglomeration and formation of floes by bridging between negatively charged particles. The authors also suggest that the “patch charge” mechanism may apply to the small and basic proteins like MOCPs. In a solution, positively charged (pI above 9.6) and low molecule weight proteins (≈6.5 kDa), bind to parts of the surfaces of negatively charged particles. This leads to the formation of negatively and positively charged parts of particle surfaces. Because of particle collision, the interparticle saturation of the differently charged sectors and formation of floes take place. In the investigations of (Fahmi et al. 2011), the water coagulation proteins extracted from *Moringa* seeds in sodium chloride solution (MO-NaCl), have shown improvement in the coagulation efficiency. This improvement has been suggested as the possible salting-in mechanism in proteins, wherein the salt increases protein–protein dissociation, leading to increased solubility. The mechanism of water coagulation and sedimentation using MOCP is outlined in Fig. 2. The mechanism of turbidity removal from soft water by MO-NaCl is proposed to be absorption and charge neutralization, following the Freundlich adsorption model. The possible mechanism of removal of hardness from hard water MO-NaCl is adsorption, modeled by both the Langmuir and Freundlich models. MO-NaCl leads to the removal of hardness from synthetic turbid, raw, and hard water by forming net-like structures followed by the sweep coagulation mechanism for turbidity removal. Of late, a magnetic coagulant based on the *M. oleifera* seed extract is found to be potentially effective to improve water parameters based on transparent color, turbidity, and compounds with UV-254 nm absorption (Santos et al. 2016).

### Biodiesel from the seed oil of *M. oleifera*

Biodiesel is a renewable and eco-friendly alternative to conventional non-renewable fossil petrodiesel fuel. Biodiesel refers to long-chain alkyl (methyl, ethyl, or propyl) esters made by chemically reacting lipids of vegetable oil and animal fat. The vegetable oils obtained from cottonseed, *Moringa*, palm, peanut, rapeseed, soybean, and sunflower have been successfully used in biodiesel preparation. Biodiesel production from palm, peanut, rapeseed, soybean, sunflower, and other conventional food sources has a strain of global food insecurity, price, and availability (Tenenbaum 2008). Thus, in the last decade, biodiesel production from less familiar and unconventional oils including *Jatropha*, *Moringa*, *Pongamia*, and tobacco have received greater attention (Rashid et al. 2008; Kivevele and Huan 2015). A survey conducted on 75 Indian plant species having 30 % or more fixed oil in their seed/kernel have concluded that fatty acid methyl esters (FAMEs) of MO seed oil meet all the main specifications of biodiesel standard of Germany, Europe, and the United States (US) (Azam et al. 2005). *M. oleifera* seeds contain 33–41 % (w/w) oil, known as “ben oil”, because of the contents of behenic acid (C_22_, docosanoic acid, ≈7 % w/w), which possesses significant resistance to oxidative degradation (Rashid et al. 2008). On account of the significant high content of monounsaturated fatty acids in the form of oleic acid (C18:1, 72.2 %), *Moringa* seeds oil is a potential candidate for biodiesel production. The fuel properties are mainly dependent on the fatty acid (FA) composition, wherein C16 and C18 monounsaturated FAMEs are ideal components to achieve an adequate balance between oxidative stability and cold flow properties of biodiesel (Knothe 2008). Thus, and FA profile can be employed as a screening tool for selection of crops for biodiesel (Moser and Vaughn 2012). In recent times, Azad et al. (2015), reviewed the prospect of *M. oleifera* (MO) seed oil as a sustainable biodiesel fuel and concluded that MO is one of the prospective industrial crops for biodiesel production in Austria.

### Properties of *M. oleifera* methyl esters

The cetane number of the *M. oleifera* methyl ester (MOME; 67.07) meets the minimum requirement of biodiesel standards of American Society for Testing and Materials (ASTM) International (ASTM D6751; 47) and European Committee for Standardization (EN 14214; 51). The properties of *Moringa oleifera* methyl esters (MOMEs) are summarized in Table 3. Using the Ignition Quality Tester™ (IQT™), the cetane numbers of methyl oleate (C18:1), methyl palmitate (C16:0), and methyl stearate (C18:0) were recorded as 59.3, 85.9, and 101, respectively. The high cetane number of MOME was explained considering the presence of other saturated FAMEs, including Eicosanoic acid (C20:0) and Docosanoic acid (C22:0). The MOME accounted for the highest cetane numbers for a biodiesel fuel (Rashid et al. 2008). The kinematic viscosity of MOME at 40 °C (4.83 mm^2^/s), was also under acceptable limits of the ASTM D6751 (1.9–6.0 mm^2^/s) and EN 14214 (3.5–5.0) biodiesel standards (Rashid et al. 2008). Also, the lubricity (ball wear scars of 135 and 138.5 μm) was well below the maximum values prescribed in the petrodiesel standards of ASTM D975 and EN 590. The maximum acceptable limits of lubricity in biodiesel are not specified in ASTM D975 and EN 590. The oxidative stability of MOME (3.61 h) was meeting the minimum value of ASTM D 6751 (3 min). However, this value was significantly lower than the minimum prescribed in the EN 14214 standard, which is 6 h. Authors suggested that the degradation and removal of antioxidants during transesterification and the subsequent purification process might have caused the decrease in the oxidative stability of MOME, compared to *M. oleifera* oil. Large numbers of studies have shown that it is difficult to prevent oxidation in biodiesel entirely, and it can only be delayed. In this regard, different strategies have been proposed to delay the oxidation, which includes, storage conditions, managing the impurities, fatty acid composition, and the addition of antioxidants in the biodiesel (Pullen and Saeed 2014).

**Table 3:** Physico-chemical properties of *Moringa oleifera* methyl esters (MOME) and their blends Data from Mofijur et al. (2014)^a^, Rashid et al. (2011)^b^ and da Silva et al. (2010)^c^

Properties	Units	Diesel^a^	MOME-1^a^	MOME-2^b^	MOME-3^c^	MB5^a^	MB10^a^	ASTM D6751	EN 14214
Dynamic viscosity	Pa s	2.69	4.34	–	–	2.81	2.94	–	–
Kinematic viscosity at 40 °C	mm^2^/s	3.23	5.05	4.8	5.4	3.39	3.55	1.9–6	3.5–5
Kinematic viscosity at 100 °C	mm^2^/s	1.24	1.84	–	–	1.3	1.36	–	–
Density at 20 °C	kg/m^3^	827.2	869.6	875 (25 °C)	883	829.6	830.6	–	860–900
Flash point	°C	68.5	150.5	162	–	74	79.5	130 min	120 min
Cloud point	°C	8	19	18	–	7	7	–	–
Pour point	°C	0	19	17	–	3	3	–	–
Cold filter plugging point	°C	5	18	17	–	6	6	–	–
Calorific value	MJ/kg	45.3	40.05	–	–	45.03	44.75	–	–
Iodine value	g I/100 g	–	77.5	–	74	–	–	–	120 max
Saponification value	–	–	199	–	–	–	–	–	–
Oxidation stability	h	–	26.2	3.52	–	–	–	3	6
Cetane number	–	48	56.3	67	–	–	–	47 min	51 min
Lubricity	HFRR; μm	–	–	139	–	–	–	–	–
Sulfur content	%	–	–	0.0124	–	–	–	0.05 max	–
Ash content	%	–	–	0.01	–	–	–	0.02 max	0.02 max
Acid value	mg KOH/g	–	–	0.38	–	–	–	0.50 max	0.50 max
Copper strip corrosion	50 °C <comma> 3 h	–	–	1	–	–	–	No. 3 max	No. 1 min
Higher heating value	MJ/kg	–	–	45.28	–	–	–	–	–
Methanol content	%	–	–	0.165	–	–	–	–	0.2 max
Free glycerin	%	–	–	0.012	–	–	–	0.020 max	0.020 max
Total glycerin	%	–	–	0.196	–	–	–	0.240 max	0.250 max

MB5 and MB10: MOME in 5 and 10 % (v/v) blend

da Silva et al. (2010) characterized MO oil of the Brazilian genotype for biodiesel production. The productivity of the Brazilian genotype was 45 tons of pods per hectare. The oil yield was 39 % from kernels, primarily composed of unsaturated fatty acids (81 %). The density of MOME (883 kg m^−3^ at 20 °C) was within acceptable ranges of EN 14214 specifications (860–900 883 kg m^−3^ at 20 °C). However, the kinematic viscosity value (5.4 mm^2^ s^−1^) was slightly higher than the maximum value specified in EN 14214, and methyl esters prepared from the MO genotypes of Pakistan (Rashid et al. 2008). The iodine value (IV; 74) obtained for the MOME was well below the maximum limits of EN 14214 specifications (120). This suggested that MOME could present a suitable degree of oxidative stability. In another study, the MOMEs prepared from the genotypes of Pakistan showed major characteristics in the acceptable range prescribed in ASTM D 6751 and EN 14214 specifications (Rashid et al. 2011). These include, density (875 kg m^−3^ at 25 °C), kinematic viscosity (4.80 mm^2^/s at 25 °C), flash point (162 °C), higher heating value (45.28 MJ/kg), cetane number (67), acid value (0.38 mg KOH/g), sulfur content (0.012 % w/w), ash content (0.010 % w/w), and water content (<0.01 % w/w). In a comparative study, the physicochemical properties of MOME in 5 and 10 % (v/v) blends (MB5 and MB10, respectively) with palm-oil blends (PB5 and PB10) and diesel fuel (B0) were evaluated in a multi-cylinder diesel engine at various speeds and under full- and half-load conditions (Mofijur et al. 2014). The engine performance indicated that the PB5 and MB5 fuels produced 1.38–2.27 % lower brake powers (BP) and 0.69–2.56 % higher brake-specific fuel consumption (BSFC) values compared to diesel fuel. These results are attributed to the higher density, viscosity, and the lower energy content of these biodiesel blends, compared to diesel. In engine emissions, PB5 and MB5 fuels reduced carbon monoxide emission by 13.17 and 5.37 % as well as hydrocarbon emission by 14.47 and 3.94 %, but slightly increased nitric oxide emission by 1.96 and 3.99 %, respectively, compared with B0. The overall performance of MB5 and MB10 was comparable with BP5, B10, and B0. Therefore, the authors suggested that these blends could replace diesel fuel in unmodified engines, as a green alternative, to reduce the global demand for fossil fuel and exhaust emissions into the environment.

### Summary and future perspective

*M. oleifera* is established as a rich source of nutritionally important phytoconstituents, which have the potential to develop functional food and nutraceuticals. However, in this regard, detailed in vitro and in vivo evaluations of bioavailability and biological activities are required to allow rational and proper recommendations of phytoconstituents. Also, as MO is becoming popular as an important food commodity in view of its current status as the “natural nutrition of the tropics”, there is ample scope for nutritional characterization of various traditional cultivars and genotypes. The high genetic diversity (variability) found in the MO cultivars and genotypes is directly linked to the higher levels of biodiversity, which is potentially beneficial for food security, productivity, and ecological sustainability. This significant variability can also be utilized in breeding programs to produce high-yielding and nutritionally rich cultivars with improved adaptations to adverse climatic conditions. Currently in India and other tropical and sub-tropical countries, major breeding programs are ongoing for the development of fast growing, disease resistant, and high pod yielding cultivars of MO. In this regard, the Bhagya (KDM-1) cultivar has been recently developed in India, and it is gaining popularity because of its high yield pod-yielding potential. Similarly, in future, attention is required to be paid to the development of cultivars for higher foliage yield, as the foliage is the richest source of carotenoids, ascorbic acids, glucosinolate and other bioactives, compared to other edible parts. Elicitors (abiotic and biotic) and signaling molecules, such as, methyl jasmonate and salicylic acid were studied for the enhancement of carotenoids and tocopherols in the foliage of *M. oleifera*. This approach could be further studied for the enhancement of other bioactives in MO for improved health benefits.

Cationic proteins from MO seeds have been seen as sustainable materials for water and wastewater treatment, to remove the turbidity, toxic metal ions, and microbial species from water. These proteins are capable of eliminating pollutants even at minor doses without developing hazardous sludge, which makes its application eco-friendly, economical, and feasible on a large scale. The coagulation efficiency is similar to chemical coagulants for high turbidity waters. However, its effectiveness is reduced for low turbidity waters. Despite the fact that various coagulation mechanisms have been proposed, the molecular basis of its coagulant and antimicrobial activity is not established.

*M. oleifera* seed oil is of low-cost and the most readily available source of biodiesel (methyl esters). The MOME prepared from seed oil exhibits a high cetane number and also meets the other major specifications of the biodiesel standard, except the oxidative stability requirement in the ASTM D6751 standard, which prescribes a minimum of 6 h. Different indices were developed for the prediction of oxidative stability of biodiesel fuel. Similarly, diverse strategies have been proposed to delay the oxidation in biodiesel fuel. However, these indices and strategies are not efficient and need further detailed investigations. Thus, with novel strategies, biodiesel production technologies should be optimized for the preservation of natural antioxidants in the fuel. The emission characteristics of MOME blends have been shown to reduce the carbon monoxide and hydrocarbon emission significantly, but it is slightly increased in nitric oxide. Limited studies are available on these emission characteristics. Therefore, further studies need to be performed on engine performance and emission by applying different combustion strategies, to get conclusive results. Overall, MO is emerging as one of the prospective industrial crops for sustainable biodiesel production in tropical and subtropical countries.
